# 基于金属有机骨架萃取剂的一步式快速固相萃取检测饮料中胭脂红

**DOI:** 10.3724/SP.J.1123.2021.01024

**Published:** 2021-12-08

**Authors:** Rong HUANG, Lei CHENG, Yushi XIAO, Qiang CAO, Na LIU, Shiheng CHEN, Lidong WU

**Affiliations:** 1.北京工商大学, 北京市食品添加剂工程技术研究中心, 食品营养与人类健康北京高精尖创新中心, 北京 100048; 1. Beijing Engineering and Technology Research Center of Food Additives, Beijing Advanced Innovation Center for Food Nutrition and Human Health, Beijing Technology and Business University (BTBU), Beijing 100048, China; 2.中国水产科学研究院, 农业农村部水产品质量安全控制重点实验室, 北京 100141; 2. Key Laboratory of Control of Quality and Safety for Aquatic Products, Ministry of Agriculture and Rural Affairs, Chinese Academy of Fishery Sciences, Beijing 100141, China; 3.上海海洋大学水产与生命学院, 上海 201306; 3. College of Food Science and Technology, Shanghai Ocean University, Shanghai 201306, China; 4.中核集团北京化工冶金研究院, 北京 101149; 4. Beijing Research Institute of Chemical Engineering and Metallurgy, China National Nuclear Corporation (CNNC), Beijing 101149, China

**Keywords:** 高效液相色谱, 一步式快速固相萃取, 金属有机骨架材料, 胭脂红, high performance liquid chromatography (HPLC), one step rapid solid phase extraction, metal-organic frameworks (MOFs), new coccine

## Abstract

胭脂红是一种广泛应用的偶氮类色素,我国对其在食品中的添加量有严格规定。由于食品基质的复杂性,针对这类痕量色素很难建立一种灵敏度高、富集性强且高效便捷的检测方法。研究利用卟啉环中的羧基与金属锆离子的配位作用制备了一种高比表面积的金属有机骨架材料(PCN-222),并将纳米材料填入注射式固相萃取装置中,以简化前处理过程,用于快速萃取饮料中胭脂红色素。采用多种表征手段研究了PCN-222纳米材料的形貌、结构及性能。较高的比表面积和静电作用共同决定了新型萃取剂对胭脂红色素具有极强的富集性能。通过优化萃取剂用量、样品溶液pH值、洗脱条件和洗脱剂体积等萃取条件,使目标物实现瞬时吸附,样品前处理时间缩短至5 min。在最佳条件下,该方法可使样品在高、中、低3个水平的加标回收试验中的回收率达到99.5%~109.4%,相对标准偏差小于3%,方法的检出限为0.1 μg/L(*S/N*=3),定量限为0.3 μg/L(*S/N*=10)。此外,针对所合成的材料进行萃取-解吸后发现,PCN-222在重复使用4次后仍保持90%以上的萃取能力,表明材料具备可循环性。该方法通过制备的新型金属骨架材料建立了一种高效、便捷、绿色环保的前处理技术,极低的检出限、较高的准确度和较好的重复性表明方法适用于饮料中痕量色素胭脂红的富集和检测。新型固相萃取技术的开发为食品安全检测提供了新思路。

## Abstract (en)

New coccine is an azo pigment that is widely used in food. To mitigate potential health issues arising from excessive consumption, China has issued provisions on the allowed addition limit of new coccine in food. Currently, there are certain difficulties with establishing detection methods for such trace pigments in foods; for example, preprocessing is complex and time-intensive. In addition, the low content of the target substance in the sample could be disturbed by food matrix, resulting in poor detection sensitivity. Metal organic frameworks (MOFs), as a novel class of highly efficient adsorbents, have attracted increasing attention because of their stability and large specific surface area. MOFs are porous coordination crystal structures that connect metal clusters with organic ligands via coordination. Owing to their molecule-sized pores, MOFs can be used in various fields such as adsorption, catalysis, and drug dispersion. However, at the same time, their ultra-high specific surface area also leads to ultra-low weight of the material itself; this makes it difficult to collect the material even under high-speed centrifugation. In this study, a MOF material (PCN-222) with a high specific surface area was prepared by the coordination of the carboxyl group in the porphyrin ring and metal zirconium ions. To simplify pretreatment, the nanomaterials were filled into an injection solid phase extraction device for the rapid extraction of new coccine pigments from beverages. The morphology, structure, and properties of the PCN-222 nanomaterials were studied by transmission electron microscopy, particle size analysis, X-ray single-crystal diffraction, infrared spectroscopy, and ultraviolet spectroscopy. The specific surface area of the synthesized material was 979 m^2^/g. A high specific surface area was conducive to the adsorption of trace target compounds. The surface charge of the material could be controlled by adjusting the pH value of the solution, which was beneficial to the selective adsorption and desorption of ionic pigments. The *π-π* interaction between the benzene ring of the porphyrin ring and the benzene ring of the azo pigment also promoted extraction. Thus, the extractant exhibited strong enrichment performance for the new coccine anionic pigment. The solid phase extraction conditions were optimized, and it was found that saturated adsorption capacity was achieved by filling 3 mg of extractant. The effect of pH on adsorption was also explored; the adsorption effect was the best at pH 3. In the desorption experiment, *N*,*N*-dimethylformamide at pH 11 was conducive to better elution of the target. Further elution volume studies showed that maximum recovery could be achieved by adding 3 mL of eluent. Subsequently, the sample pretreatment time was reduced to 5 min. The enriched sample was separated using a Zorbax eclipse XDB-C18 column (250 mm×4.6 mm, 5 μm), eluted with an ammonium acetate-methanol solvent system, and detected at 254 nm. Under the optimum conditions, the recoveries of the samples at high, medium, and low levels reached 99.5%-109.4%, and the relative standard deviation was less than 3%. The limit of detection (LOD, *S/N*=3) of this method was 0.1 μg/L and the limit of quantification (LOQ, *S/N*=10) was 0.3 μg/L. In the actual sample detection experiment, the detection signal of new coccine in the sample was amplified by solid phase extraction to achieve enrichment. In addition, the extraction capacity of PCN-222 remained higher than 90% after four uses, and the synthesized material could be recycled. The high precision and low detection limit indicate that the method is suitable for the enrichment and detection of trace carmine in beverages. The findings of this study will aid in the development of a new solid phase extraction technology for food safety evaluation.

为了增加食物的视觉美感,合成色素常常被添加到食品中。胭脂红是一种色泽鲜艳的偶氮类色素,其在食品中的使用范围和限量标准在GB 2760-2014《食品安全国家标准 食品添加剂使用标准》中有严格规定,饮料类食品中胭脂红不能超过0.05 g/kg。但仍有不良商家为了追求利益而忽视这些标准。过量的偶氮类色素被人体摄入后,会对肝脏和神经系统造成危害,甚至会增加靶器官致畸、致癌、致突变的风险^[1,2,3]^。因此,建立一套简单、高效的针对食品中胭脂红含量的检测体系,对食品质量监管及消费者身体健康有重大意义。

在建立检测色素分析方法的过程中,研究者相继开发出多种应用于食品着色剂含量的成熟检测手段,如薄层色谱法^[4]^、反向高效液相色谱法^[5]^、离子对液相色谱法^[6]^、液相色谱-串联质谱法^[7,8]^。因为食品基质较为复杂且色素含量一般较低,高效的前处理技术成为精确检测的关键。目前,合成色素常用的前处理方法包括液液萃取^[9,10]^、固相萃取^[11,12,13]^、液相微萃取^[14]^、分子印迹固相萃取^[15]^等。其中固相萃取技术应用最为广泛,其发展与吸附剂的性能密切相关。金属有机框架(MOFs)作为一种新型高效吸附剂逐渐受到广泛关注^[16,17,18,19]^。MOFs是通过配位方式将金属团簇与有机配体相互连接的多孔配位晶体结构。金属有机框架具有分子尺寸的孔洞,使其在吸附、催化、药物分散等不同领域有多种应用^[20,21,22,23,24,25,26,27,28,29]^。但是MOFs材料在实际应用中也存在一定局限性,例如超高的比表面积会导致MOFs材料本身质量超轻,即使高速离心也很难收集^[30]^,因此MOFs纳米材料在实际应用中受到一定限制,无法在各个领域广泛推广。

本工作将新型纳米材料PCN-222应用于固相萃取技术中,即合成的MOFs作为萃取剂添加到注射式固相萃取针筒内,利用材料同目标物胭脂红分子之间存在的静电和*π-π*作用,实现瞬时性高效萃取。同时,PCN-222的可重复利用性可以减少其向环境中的排放次数,以满足绿色化学的要求。与广泛应用的液液萃取和固相萃取相比,本方法的开发节省了大量前处理时间和实验成本,为食品安全快速检测提供了高效的技术手段。

## 1 实验部分

### 1.1 仪器、试剂与材料

JEM-2010透射电子显微镜(日本电子光学公司), Nicolet IS5傅里叶变换红外分光光度计(美国赛默飞公司), SPECORD 200 PLUS紫外/可见分光光度计(德国耶拿公司), Zetasizer Nano ZS粒度分析仪(英国马尔文科技公司), LC-20AT高效液相色谱仪(日本岛津公司)。

*N*,*N*-二甲基甲酰胺(DMF)和苯甲酸(BA)购于上海阿拉丁试剂公司;四(4-羧苯基)卟啉(TCPP)和胭脂红购于日本TCI试剂公司;八水氯化锆(ZrOCl_2_·8H_2_O)购于北京InnoChem科技公司;氨水(NH_3_·H_2_O)和乙醇(C_2_H_5_OH)购于上海麦克林生化科技公司,甲醇购于上海安谱公司;18.2 MΩ·cm超纯水通过Milli-Q(美国Millipore公司)超纯水仪制得。

### 1.2 标准溶液的配制

精确称取胭脂红粉末10 mg,用水溶解后转移至10 mL棕色容量瓶中,稀释至刻度,得到1 g/L的标准储备液,于-4 ℃储存,使用时用超纯水梯度稀释成质量浓度分别为50、100、200、300、500、1000、5000、10000 μg/L的系列标准溶液。

### 1.3 PCN-222的合成

取150 mg ZrOCl_2_·8H_2_O、2.8 g苯甲酸、50 mg TCPP和50 mL DMF溶液(含1%超纯水,下同),置于圆底烧瓶中,经2~3 min的超声混匀后,转移至90 ℃油浴锅内加热搅拌4 h。最后,以13000 r/min离心30 min,收集产物,用DMF溶液离心洗涤3次,然后再分散在甲醇中,得到深紫色产物PCN-222。

### 1.4 样品前处理

取3 mg PCN-222作为萃取剂,加入到含有筛板的注射器中(如图1所示),以甲醇为匀浆液通过湿法装柱。预处理活化过程:5 mL甲醇活化,5 mL水淋洗后上样。

**图1:**
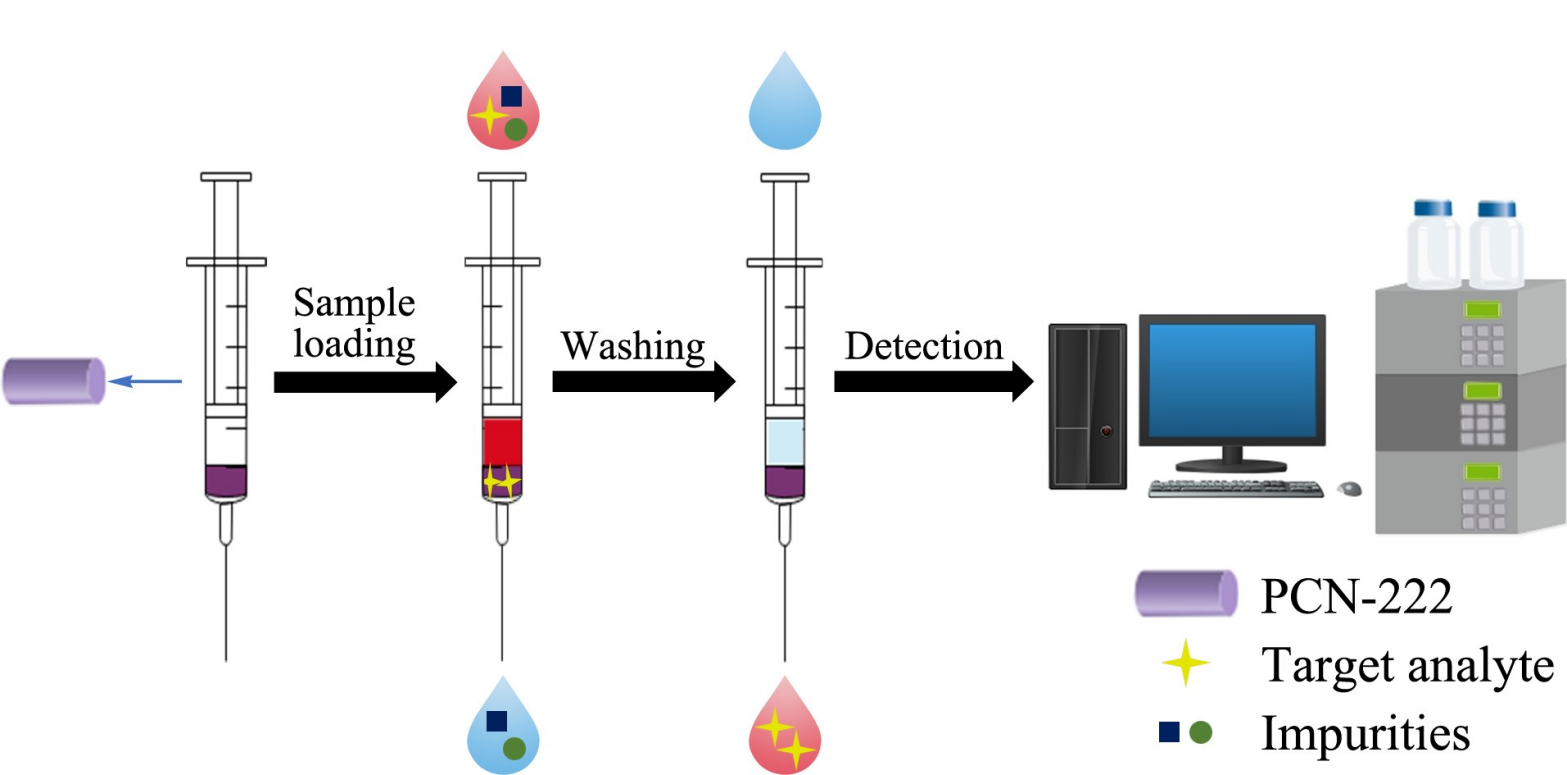
微型注射式固相萃取流程图

分别取1 mL饮料样品,用超纯水稀释至4 mL,通过盐酸调节样品溶液pH值至3,然后将稀释后的样品加入到注射器中进行上样。通过压力使目标分析物流过并被截流在萃取剂上。加入3 mL洗脱液DMF溶液(用NH_3_·H_2_O调节pH值至11)。缓慢推动柱塞,使染料从吸附剂上解吸下来,收集洗脱液,于45 ℃氮气吹干,然后用1 mL水复溶,用0.45 μm滤膜过滤后,供HPLC分析。

### 1.5 仪器条件

色谱柱:ZORBAX Eclipse XDB-C18色谱柱(250 mm×4.6 mm, 5 μm);柱温:40 ℃;流动相A:乙酸铵溶液(0.02 mol/L);流动相B:甲醇;流速:1 mL/min。梯度洗脱程序:0~3 min, 5%B~35%B; 3~7 min, 35%B~100%B; 7~10 min, 100%B; 10~15 min, 100%B~5%B。进样量:10 μL;检测波长:254 nm。

## 2 结果与讨论

### 2.1 PCN-222的表征

用透射电镜观察制备好的PCN-222的形貌(见图2)。由TEM图像可以看出,合成的MOFs材料为棒状结构,是一种形貌均一的纳米材料,沿长轴方向延伸。根据粒径分布图3a所示,所合成的大部分PCN-222粒径分布在500~700 nm之间,这与透射电镜图所显示的棒状结构的直径大小基本吻合。

**图2:**
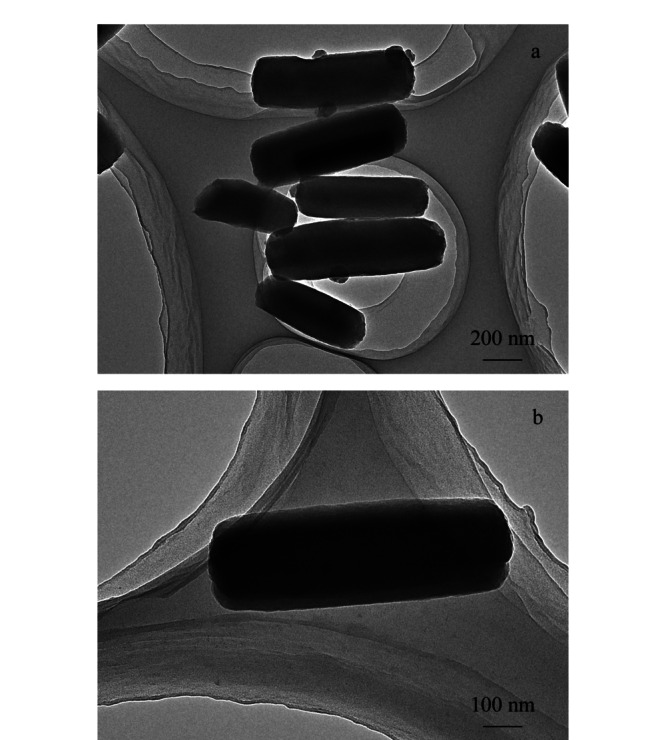
PCN-222的透射电镜图

**图3:**
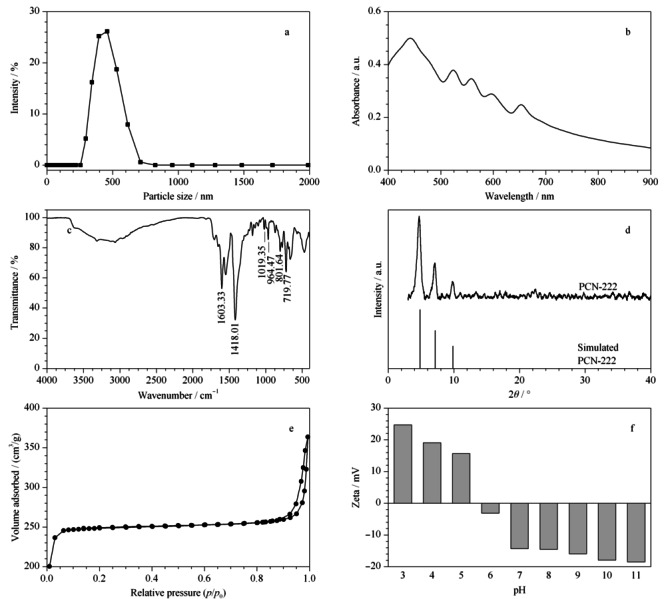
PCN-222的(a)粒径分布图、(b)紫外可见光谱图、(c)红外光谱图、(d)X射线晶体衍射图、(e)N_2_吸附/脱附曲线和(f)Zeta电位分布情况关系图

对所合成的材料进行紫外可见光谱分析,由图3b可知,卟啉型MOFs的典型吸收峰。PCN-222的主吸收峰在435 nm, 4个Q带吸收峰分别位于524、558、596和653 nm处。

通过红外光谱进一步分析样品组成,结果如图3c所示,在719.77、801.64和964.47 cm^-1^处存在卟啉环的伸缩振动峰;在1019.35、1418.01和1603.33 cm^-1^处存在苯环骨架的吸收峰。

为了准确分析样品的晶体结构,进一步对PCN-222进行X射线衍射(XRD)表征(见图3d)。可以看到,所合成材料XRD曲线峰位置与模拟的PCN-222曲线峰位置可一一对应,表明晶体结构完整。

对材料进行N_2_吸附-脱附表征以验证所合成的材料具备较强的吸附能力,材料的BET比表面积为979 m^2^/g,较大的比表面积适用于食品中痕量色素的吸附(见图3e)。

Zeta电位可以对材料表面电荷情况进行探究,以考察静电作用对萃取性能的影响。如图3f所示,随溶液内pH值变化,PCN-222表面电位逐渐发生变化,当pH<6时,由于氢离子在材料表面积累而呈正电荷;当pH>6时,MOFs表面整体呈负电荷,这是由于OH^-^在其表面积累。结果表明,所合成的材料具有较大的正负电位跨度,通过控制其pH值就可以促进材料选择性吸附和分离,证明其是一种具有电荷可调性的MOFs材料。

### 2.2 固相萃取条件优化

为了确定PCN-222萃取剂对目标物富集净化的最佳条件,本工作对影响固相萃取效率的诸多因素进行了考察,包括萃取剂用量、样品pH值、洗脱剂种类和洗脱剂pH值等,考虑到样品基质的影响,选取在不含目标物的加标(10 mg/L)样品基质中进行。

2.2.1 萃取剂用量

合适的萃取剂用量在保证柱容量的同时还可以节约实验成本。因此本工作首先对PCN-222吸附剂的用量进行了探究,考察了萃取剂用量为1~4 mg时,吸附效率的变化(见图4a所示)。结果表明,实验初期随着萃取剂用量的增加,胭脂红的吸附率逐渐升高,当用量超过3 mg后,吸附率基本不再变化。这是因为随着萃取剂的增加,反应体系内的活性位点也逐渐增加,从而对目标物的吸附能力有所提升。当材料对目标分析物的萃取达到饱和时,即使后期继续增加萃取剂的用量,吸附率也不再发生变化。因此最终选择3 mg萃取剂用量的萃取小柱进行后续研究。与其他材料相比,PCN-222作为固相萃取剂在保证最大吸附效率的同时节约了柱容量。

**图4:**
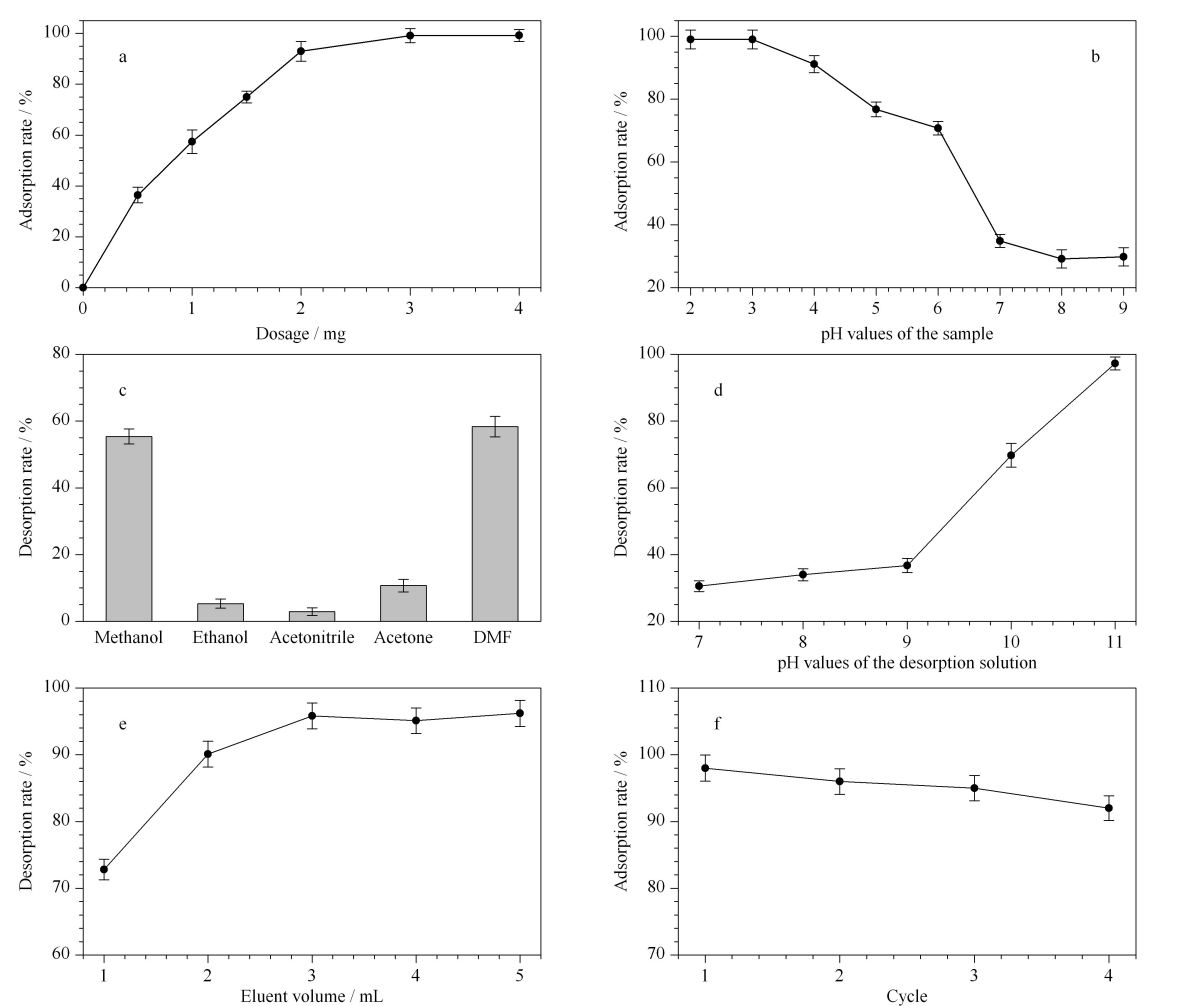
(a)吸附剂用量、(b)样品pH值、(c)不同解吸液、(d)解吸液pH值、(e)解吸体积和(f)重复利用次数对胭脂红萃取效率的影响(*n*=3)

2.2.2 样品pH值

样品溶液的pH值决定了吸附剂在溶液中所带电荷的不同,导致其与胭脂红溶液之间的作用力也不同,从而影响吸附率。因此考察溶液pH值对吸附性能的影响具有重要意义。本工作探究了样品溶液pH值分别为2~9时吸附率的变化情况,结果见图4b。样品溶液的吸附率随pH值增加而降低。在酸性条件下,正电荷逐渐在多孔材料PCN-222表面积累,对本身为阴离子色素的胭脂红的吸引力逐渐增加,考虑到材料的稳定性^[31]^,最终选择在样品溶液pH值为3的条件下进行萃取。

2.2.3 洗脱剂

不同极性的有机溶剂对化合物的洗脱能力不同,因此为了达到最高的萃取效率有必要对洗脱剂种类进行优化。本工作研究了甲醇、乙醇、乙腈、丙酮和*N*,*N*-二甲基甲酰胺这5种洗脱剂对目标分析物的解吸能力。如图4c所示,极性较高的溶剂甲醇和DMF对目标物的洗脱能力较强。同时结合油水分配系数(Log *P*)越小,化合物极性越强的规律,可以发现,由于DMF的Log *P*值最小,使得解吸效率最高。因此最终选择了解吸效果最佳的DMF溶剂作为解吸剂,但其解吸率仍没有达到100%。考虑到在碱性条件下吸附剂带负电荷,目标物在静电排斥作用下更易洗脱。因此,向有机溶剂中加入少量氨水后进行实验,回收率大大提高。为了进一步探究解吸性能与pH的关系,考察了不同pH值对解吸效率的影响(见图4d)。结果表明,随着pH值升高,解吸率逐渐升高。因此最终选用pH 11的DMF作为最佳洗脱剂。

2.2.4 洗脱剂用量

洗脱剂用量影响待测物从萃取剂上洗脱下来的程度。洗脱剂用量太少会使目标物洗脱不完全,用量太多会增加后续的工作量,不能实现富集。因此本文考察了不同体积(1~5 mL)的洗脱剂对胭脂红染料的洗脱情况,结果见图4e。当洗脱剂体积达到3 mL时,对目标物的吸附回收率达到最大且后续没有明显的增加。综合考虑实验结果与实验成本,最终确定洗脱剂最佳用量为3 mL。

### 2.3 材料的重复利用

为了能够重复利用PCN-222萃取剂,以减少实际检测成本,本实验对材料的重复利用率进行了探究。将使用过带有萃取剂的微注射式固相萃取柱经超纯水和甲醇反复洗涤后再次用于样品前处理过程,结果见图4f。研究发现,萃取剂在重复使用4次后,萃取能力虽有所下降,但仍高于90%,说明PCN-222不仅对阴离子胭脂红溶液具备高效的吸附性能,而且还可以重复利用。与其他固相萃取柱相比,本研究所开发的一步式固相萃取装置节约了检测成本,是一种绿色环保的前处理装置。

### 2.4 方法学考察

2.4.1 检出限、定量限和线性范围

在最优条件下,以3倍和10倍信噪比计算胭脂红的检出限和定量限,分别为0.1 μg/和0.3 μg/L。以质量浓度为横坐标(*x*, μg/L),对应的峰面积为纵坐标(*y*),绘制标准曲线。目标物在50~10000 μg/L范围内表现出良好的线性关系,线性方程为*y*=29.3*x*+2175.5,相关系数(*r*^2^)大于0.999。

2.4.2 准确度和精密度

通过向空白样品基质中加入高、中、低3个不同水平(0.1、1、10 μg/mL)的标准溶液进行加标回收试验,以回收率表示方法的准确度,同时测定6份平行样品。结果表明,饮料中胭脂红的加标回收率为99.5%~109.4%,相对标准偏差(RSD)为0.55%~2.4%。表明本方法在低浓度和高浓度下均具备良好的准确度和精密度,满足实际样品的测定需求。

### 2.5 实际样品分析

将本方法应用于市场上购买的几种饮料中胭脂红的含量分析,结果表明饮料中均未检出胭脂红。对比研究了空白加标的维生素饮料(10 μg/mL)分别通过一步式快速萃取富集和直接应用HPLC的差异。结果如图5所示,由于样品基质背景十分复杂,加标后直接用于液相色谱检测的色谱图受杂质影响而使目标峰的响应值极低,经过微注射式固相萃取后,胭脂红的峰响应明显增高,其他杂峰消失,说明本方法对样品中胭脂红可实现富集,能够排除其他杂质的干扰,有利于样品中痕量目标物的检测。

**图5:**
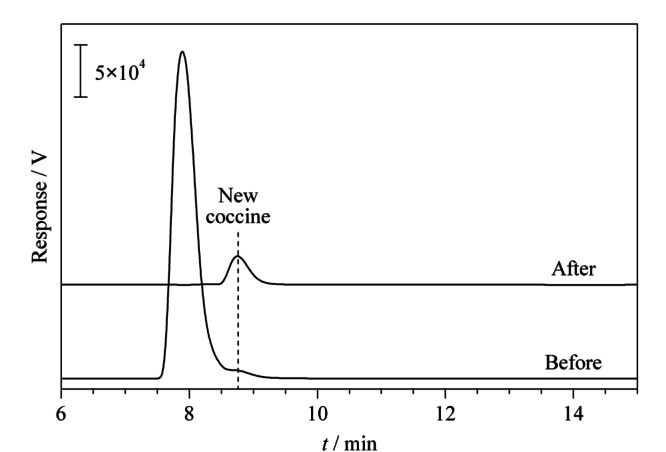
经微注射固相萃取前、后加标维生素饮料(10 μg/mL)的色谱图

### 2.6 与其他方法比较

将本方法与广泛应用在食品样品中痕量色素的检测方法进行比较(见表1)。可以看出,本方法对目标色素的检测具备较低的检出限和较好的重复性。此外,可以发现目前常用的检测方法在前处理阶段仍需花费大量的时间,本工作通过自制的微注射固相萃取技术明显简化了前处理流程,在5 min内即可完成萃取过程,在饮料等实际样品检测中具有较好的应用潜力。

**表1:** 本方法与相关文献中方法的比较

Method	Sample	Analytes	Extraction time/min	LOD/ (μg/L)	Intra-day RSD/%	Ref.
Polyamide-SPE-HPLC-UV	dried-strawberry	new coccine	>60	-	<10	[32]
LLE-UPLC-MS/MS	feed	new coccine, para red	>55	6.0-12.0	<24	[33]
Alumina N-SPE-HPLC-DAD	chili	sudan Ⅰ, Ⅱ	>105	4.2-5.6	<5	[34]
MOFs μSPE-HPLC-UV	beverages	new coccine	5	0.1	<3	this work

LLE: liquid liquid extraction; DAD: diode array detector; MOFs: metal-organic frameworks; μSPE: micro-solid phase extraction; -: no data.

## 3 结论

本工作将合成的基于金属骨架的纳米材料PCN-222作为高效萃取剂,应用于自制微注射式的固相萃取设备,在保持超高萃取效率的同时大大简化了萃取步骤。本文建立了一步式快速萃取饮料中偶氮类染料胭脂红的新检测方法,在最优的固相萃取条件下,PCN-222萃取剂展现出良好的萃取能力,并能够有效降低食品基质中杂质对液相色谱分离的影响,实现了对痕量胭脂红的富集。检测方法具备极低的检出限、较高的准确度和较好的重复性。本方法具备快速、灵敏、高效和环保等实用价值,为未来新型纳米材料与食品检测技术的交叉研究提供理论依据和应用参考。
